# Carbamylated Erythropoietin Decreased Proliferation and Neurogenesis in the Subventricular Zone, but Not the Dentate Gyrus, After Irradiation to the Developing Rat Brain

**DOI:** 10.3389/fneur.2018.00738

**Published:** 2018-09-12

**Authors:** Kazuhiro Osato, Yoshiaki Sato, Akari Osato, Machiko Sato, Changlian Zhu, Marcel Leist, Hans G. Kuhn, Klas Blomgren

**Affiliations:** ^1^Center for Brain Repair and Rehabilitation, Institute of Neuroscience and Physiology, Gothenburg University, Gothenburg, Sweden; ^2^Department of Obstetrics and Gynecology, Mie University, Tsu, Japan; ^3^Division of Neonatology, Center for Maternal-Neonatal Care, Nagoya University Hospital, Nagoya, Japan; ^4^Department of Obstetrics and Gynecology, Narita Hospital, Nagoya, Japan; ^5^Department of Pediatrics, The Third Affiliated Hospital of Zhengzhou University, Zhengzhou, China; ^6^Department of Biology, University of Konstanz, Konstanz, Germany; ^7^Department of Women's and Children's Health, Karolinska Institutet, Department of Pediatric Hematology and Oncology, Karolinska University Hospital, Stockholm, Sweden

**Keywords:** radiotherapy, pediatric oncology, late effects, immature brain, neural stem cell

## Abstract

Cranial radiotherapy for pediatric brain tumors causes progressive, debilitating late effects, including cognitive decline. Erythropoietin (EPO) has been shown to be neuroprotective and to promote neuroregeneration. Carbamylated erythropoietin (CEPO) retains the protective properties of EPO but is not erythrogenic. To study the effects of CEPO on the developing brain exposed to radiotherapy, a single irradiation (IR) dose of 6 Gy was administered to the brains of postnatal day 9 (P9) rats, and CEPO (40 μg/kg s.c.) was injected on P8, P9, P11, P13, and P15. To examine proliferation, 5-Bromo-2-deoxyuridine (BrdU) was injected on P15, P16, and P17. CEPO administration did not affect BrdU incorporation in the granule cell layer (GCL) of the hippocampus or in the subventricular zone (SVZ) as quantified 7 days after the last BrdU injection, whereas IR decreased BrdU incorporation in the GCL and SVZ by 63% and 18%, respectively. CEPO did not affect BrdU incorporation in the GCL of irradiated brains, although it was reduced even further (to 31%) in the SVZ. To evaluate the effect of CEPO on neurogenesis, BrdU/doublecortin double-positive cells were quantified. CEPO did not affect neurogenesis in non-irradiated brains, whereas IR decreased neurogenesis by 58% in the dentate gyrus (DG) but did not affect it in the SVZ. In the DG, CEPO did not affect the rate of neurogenesis following IR, whereas in the SVZ, the rate decreased by 30% following IR compared with the rate in vehicle-treated rats. Neither CEPO nor IR changed the number of microglia. In summary, CEPO did not promote neurogenesis in non-irradiated or irradiated rat brains and even aggravated the decreased neurogenesis in the SVZ. This raises concerns regarding the use of EPO-related compounds following radiotherapy.

## Introduction

Nearly one-third of all pediatric malignancies are brain tumors, and their incidence has increased over the last decades (1–3). Improved treatment protocols have considerably increased the survival of patients with such malignancies, with >80% of patients currently surviving their disease (4). Treatment strategies for pediatric malignancies are associated with late adverse effects such as perturbed growth, endocrine dysfunctions, learning difficulties, and cognitive decline (5). Children receiving radiotherapy to the CNS are at the greatest risk of cognitive decline (6), which increases with younger age at diagnosis (7, 8). Thus, ameliorating the late effects of CNS radiotherapy would greatly improve the quality of life of the increasing numbers of childhood cancer survivors. However, effective preventions, protective treatments, and rehabilitation strategies are currently unavailable in clinical practice and experimental research.

Therapeutic doses of ionizing irradiation (IR) result in increased apoptosis (9, 10) and decreased cell proliferation in neurogenic regions (11). Both the subventricular zone (SVZ) and the subgranular zone (SGZ) contain proliferating neural stem and progenitor cells and undergo neurogenesis throughout life (12–14). Thus, these regions are particularly susceptible to IR-induced apoptosis (15), and animal studies of hippocampal function have supported the hypothesis that this decrease in neurogenesis contributes to cognitive deficits experienced by patients after cranial radiation therapy (15).

Erythropoietin (EPO) has been shown to have neuroprotective properties in several animal models (16), including those of spinal cord injury (17), adult focal ischemia (18), neonatal hypoxia-ischemia (19), neonatal white matter injury (20, 21), traumatic brain injury (22), chronic autoimmune encephalomyelitis (23), and amyotrophic lateral sclerosis (24) as well as neonatal brain injury (25, 26). In addition to its neuroprotective effects, EPO affects other tissues, including the heart (27), kidneys (28), intestine (29), liver (30), and skin (31). EPO can exert protective effects via several different mechanisms, such as the attenuation of apoptosis (32), excitotoxicity (33), oxidative stress (29), and inflammation (34), as well as through angiogenesis (35). We have previously failed to demonstrate the neuroprotective effects of EPO or caspase inhibition through XIAP overexpression in neonatal mice exposed to IR (36). However, apoptosis inhibition through lithium treatment was found to reduce the morphological and behavioral damage caused by IR to the young mouse brain (9). Carbamylated erythropoietin (CEPO), in which all lysine residues are transformed to homocitrulline via carbamylation, does not bind to the classical EPO receptor but retains the tissue-protective properties without affecting erythrogenesis and hematocrit (37). CEPO binds to the β common receptor, which may be involved in its neuroprotective activity (38). Protective effects of CEPO have been shown to resemble those of EPO in various models (21, 39, 40). In addition, CEPO has been demonstrated to enhance proliferation *in vitro* (41) and *in vivo* (42) and to promote neurite outgrowth and neuronal spine formation (43).

In the present study, we tested the hypothesis that CEPO enhances proliferation in the neurogenic regions, thereby enhancing recovery after IR.

## Materials and methods

### Animals

All animal experimental protocols were approved by the Gothenburg Animal Ethics Committee of the Swedish Board of Agriculture (46-2007 and 326-09). Wistar rats were purchased from B & K Universal (Solna, Sweden).

### Irradiation procedure

IR was performed as previously described (44, 45). Briefly, a linear accelerator (Varian Clinac 600CD) with a 4 MV nominal photon energy and a dose rate of 2.3 Gy/min was used. Male rats (9-day-old) were anesthetized with the intraperitoneal administration of tribromoethanol (Sigma-Aldrich, Stockholm, Sweden) and placed in the prone position (head to gantry) on an expanded polystyrene bed. The left cerebral hemisphere of each rat was irradiated with an asymmetrical radiation field of 1 × 2 cm without divergence toward the right hemisphere. The distance from the source to the skin was approximately 99.5 cm. The head was covered with a tissue equivalent bolus material (1 cm). A single absorbed dose of 6 Gy was administered to each rat. The variation of dose within the target volume was estimated to be ±5%. The entire procedure was performed within 10 min. After IR, rats were returned to their biological dams until they were sacrificed. Sham control rats were anesthetized but did not receive IR. Using the LQ-model (46) and an α/β-ratio of 3 for late effects in the normal brain tissue, an acute exposure of 6 Gy is equivalent to approximately 12 Gy when delivered in daily 2 Gy fractions, which represents a clinically relevant dose, equivalent to that used in prophylactic cranial IR in children with acute lymphatic leukemia.

### Carbamylated erythropoietin treatment and 5-bromo-2-deoxyuridine injection

**Figure 1:**
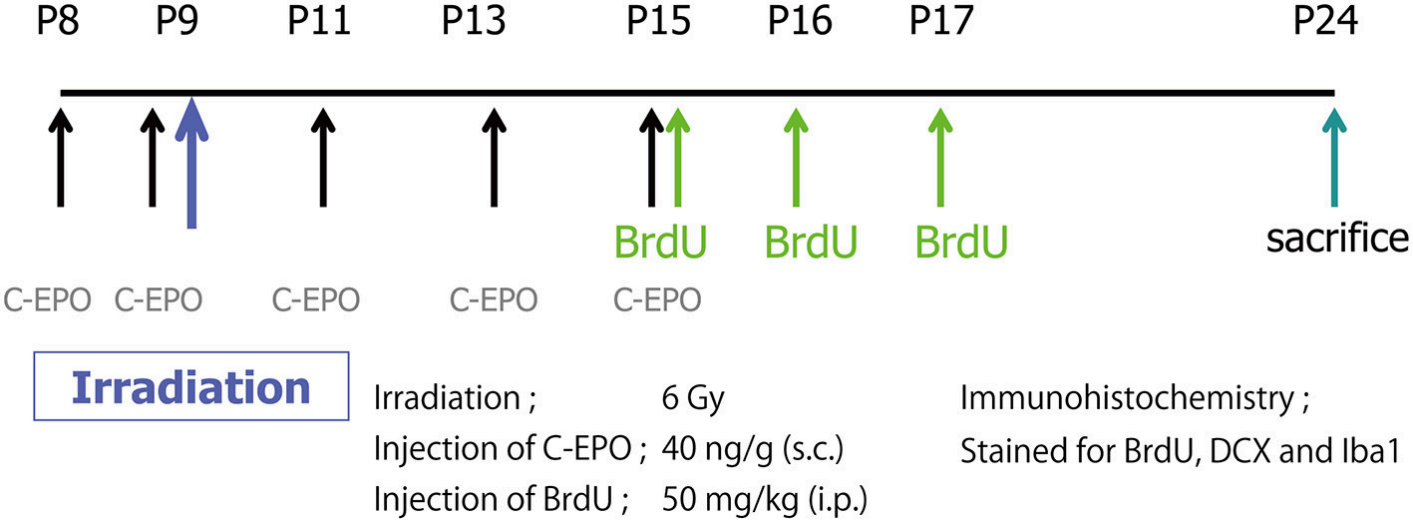
A schematic diagram of the experimental procedures. A single IR dose of 6 Gy was administered to the brains of P9 rat pups. CEPO (40 μg/kg s.c.) was administered on P8, P9, P11, P13, and P15. BrdU (50 mg/kg i.p.) was injected on P15, P16, and P17. Animals were sacrificed on P24, and their brains were processed for immunohistochemistry.

CEPO (40 μg/kg), prepared and characterized as previously described in detail (37), was subcutaneously administered on P8, P9, P11, P13, and P15. 5-bromo-2-deoxyuridine (BrdU) (1 mg/mL, dissolved in PBS; Roche, Mannheim, Germany; 50 mg/kg) was intraperitoneally injected on P15, P16, and P17. Rats were sacrificed on P24, and their brains were processed for immunohistochemistry. A schematic diagram of the experimental procedures is shown in Figure 1.

### Tissue preparation

On P24, rats were deeply anesthetized and subjected to intracardial perfusion with 0.9% NaCl followed by 5% buffered formaldehyde (Histofix, Histolab, Gothenburg, Sweden). Brains were removed and immersion-fixed in the same solution overnight at 4°C and then immersed in 30% sucrose for at least 2 days. Brains were then coronally cut at 40 μm on a sliding microtome in dry ice. Sections were stored at −20°C in a cryoprotectant solution (50% glycerol/25% glycol/25% 0.1 M phosphate buffer).

### Immunohistochemistry

Following antibodies and final dilutions were used: mouse anti-BrdU (1:500; 11170376, Roche, Mannheim, Germany), goat anti-doublecortin (DCX; 1:500; sc-8066, Santa Cruz Biotechnology, Santa Cruz, CA, USA), rabbit anti-Iba1 (1:1,000; 019-19741, WAKO Pure Chemical Industries, ltd., Osaka, Japan), donkey anti-mouse-Alexa488 (1:1,000; A21202, Invitrogen Corporation, Carlsbad, CA, USA), and donkey anti-goat-Alexa555 (1:1,000; A-21432, Invitrogen Corporation).

Immunoperoxidase detection of BrdU was performed as previously described (45). Briefly, sections were rinsed in Tris-buffered saline [TBS; 0.1 M Tris-HCl (pH 7.4) in 0.9% NaCl], treated with 0.6% H_2_O_2_ in TBS for 30 min, incubated with 50% formamide/2 × SSC (0.3 M NaCl, 0.03 M sodium citrate) at 65°C for 2 h, rinsed with 2 × SSC for 5 min, incubated with 2 N HCl at 37°C for 30 min, and rinsed with 0.1 M boric acid (pH 8.5) for 10 min. Incubation with 3% donkey serum and 0.1% Triton X-100 in TBS (TBS++) for 30 min was followed by overnight incubation with mouse anti-BrdU. After several rinses in TBS 2 × 10 min, sections were incubated with donkey anti-mouse-biotin (1:1,000; Vector Laboratories, Burlingame, CA, USA) for 1 h and then in avidin–biotin–peroxidase complex (Vectastain ABC Elite kit, Vector Laboratories), followed by peroxidase detection for 5 min (0.25 mg/ml DAB, 0.01% H_2_O_2_, 0.04% NiCl). For Iba-1 staining, sections were treated with 0.6% H_2_O_2_ in TBS for 30 min. After rinsing, sections were blocked with TBS++ and incubated with rabbit anti-Iba1 at +4°C for 24 h in TBS++, followed by 1 h at room temperature with donkey anti-rabbit-biotin (1:1,000, Vector Laboratories).

Double immunofluorescence for BrdU and DCX was performed as follows: sections were incubated with 2 N HCl at 37°C for 30 min and rinsed with 0.1 M boric acid (pH 8.5) for 10 min. After several rinses with TBS, sections were incubated with TBS++ for 30 min, followed by a primary antibody cocktail, including mouse anti-BrdU and goat anti-doublecortin, in TBS++ at +4°C for 24 h. The sections were then rinsed with TBS 3 × 10 min, incubated with a cocktail of fluorochrome-labeled secondary antibodies for 2 h, rinsed again with TBS, and mounted on glass slides.

### Cell counting

BrdU-positive cells were counted throughout the SVZ and SGZ of the dentate gyrus (DG) of the hippocampus, and Iba1-positive cells were counted throughout the SVZ and granule cell layer (GCL), hilus, and molecular layer (ML). The numbers were expressed as densities, divided by the area of the SVZ, GCL, hilus, or ML or the length of the SGZ. All counts and measurements were performed using Stereo Investigator ver. 6 (MBF Bioscience, Williston, VT, USA).

### Statistics

Results are presented as mean ± S.E.M. Statistical analysis was performed using one-way ANOVA followed by Fisher's *post-hoc* test using SPSS 17.0 (SPSS Inc., Chicago, IL, USA). p < 0.05 was considered significant.

## Results

### Impact of CEPO on proliferation after IR

**Figure 2:**
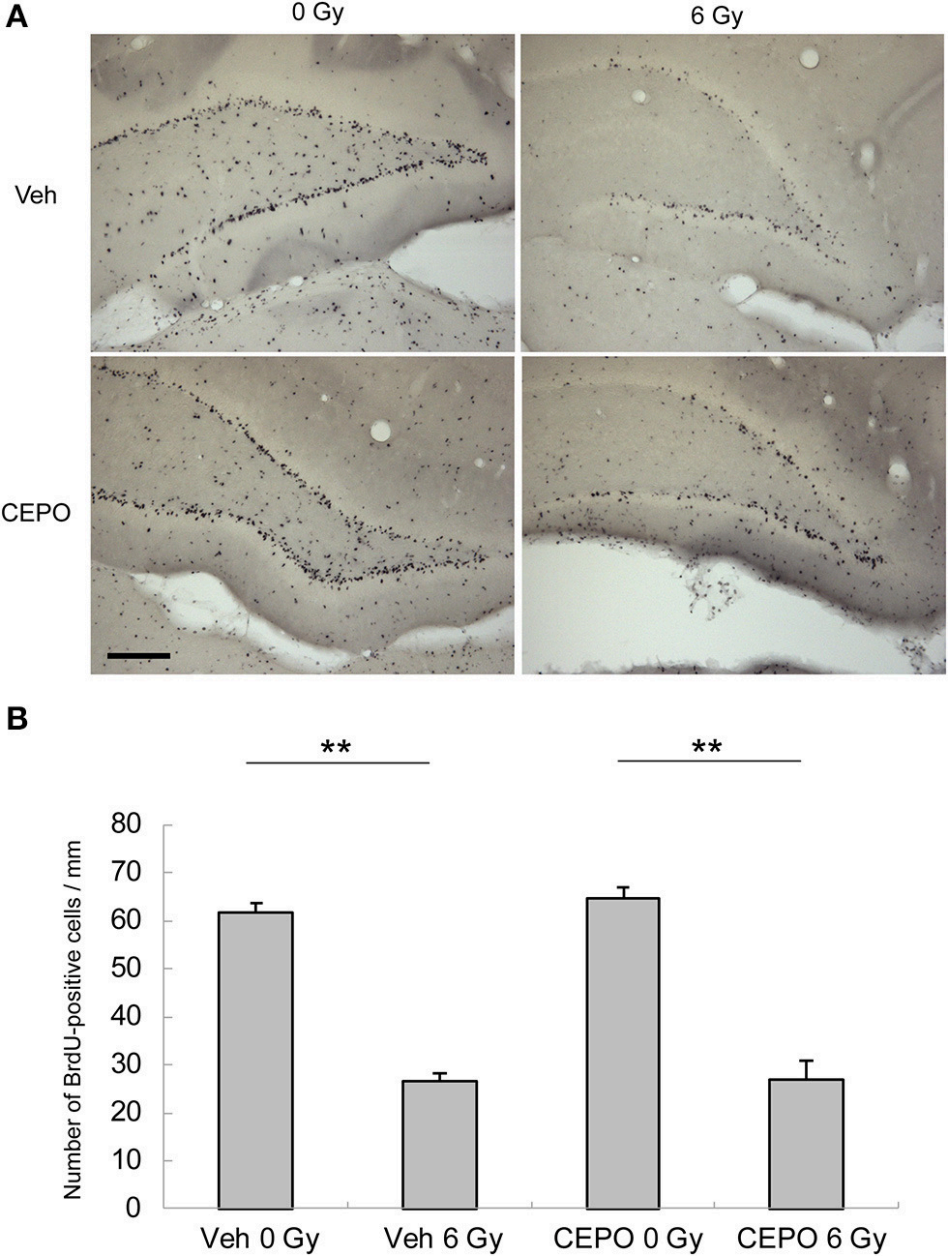
Proliferation and survival in the subgranular zone (SGZ) of the dentate gyrus (DG). **(A)** Representative microphotographs of the dentate gyrus (DG) of the hippocampus stained for BrdU in vehicle-treated rats (Veh) without IR (0 Gy), in vehicle-treated rats after IR (6 Gy), and in CEPO-treated (CEPO) rats without or with IR. Bar = 100 μm. **(B)** The number of BrdU-positive cells in the subgranular zone (SGZ) of vehicle-treated rats after IR (*n* = 8) was significantly lower than that of non-irradiated vehicle-treated rats (*n* = 7) (**p < 0.01). The number of BrdU-positive cells in the SGZ of CEPO-treated rats after IR (*n* = 6) was significantly lower than that of non-irradiated CEPO-treated rats (*n* = 9) (**p < 0.01). CEPO did not alter the number of BrdU-positive cells in non-irradiated or irradiated rats. Positive cells were counted as described in the Materials and methods section. Results are presented as mean ± S.E.M.

**Figure 3:**
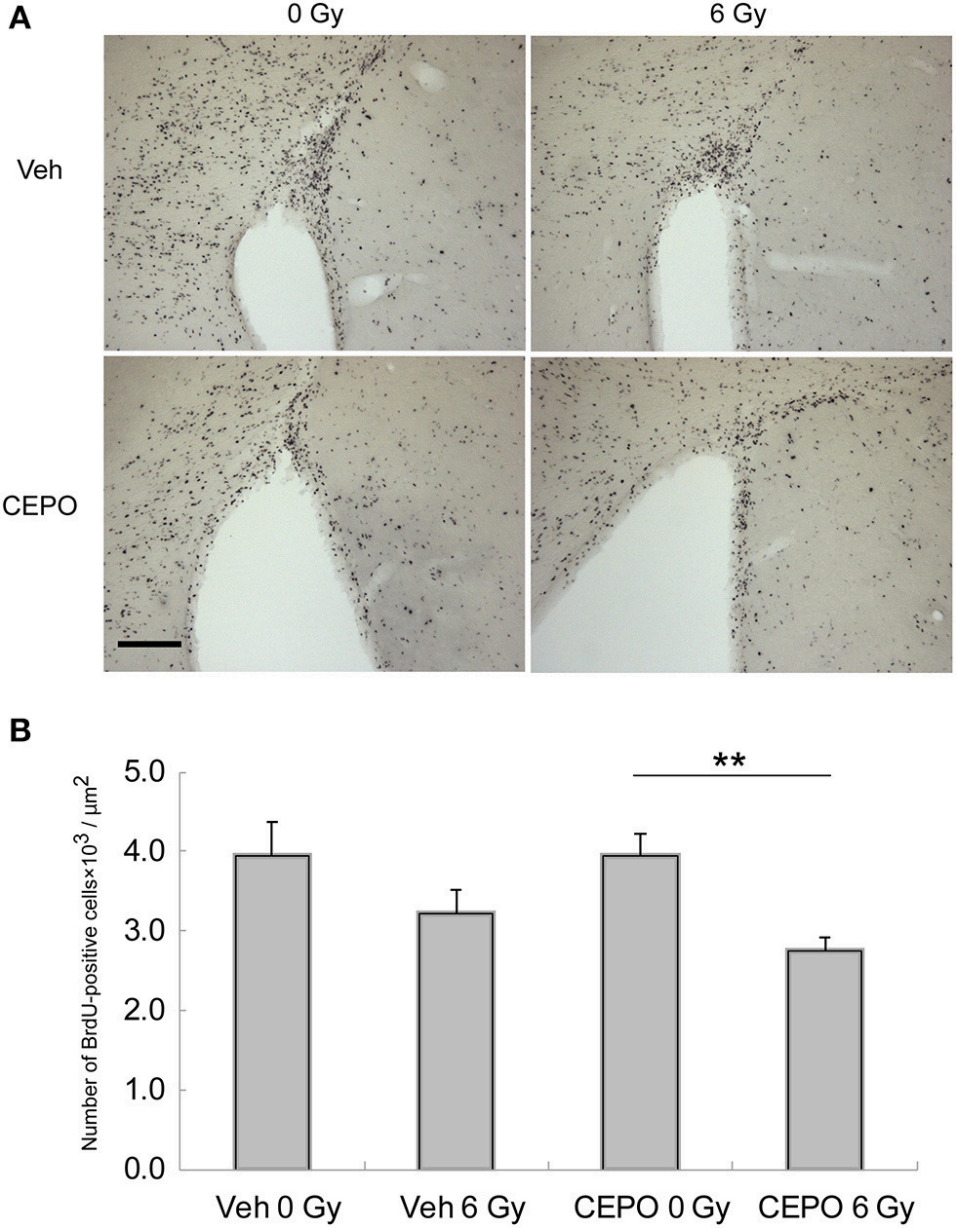
Proliferation and survival in the subventricular zone (SVZ). **(A)** Representative microphotographs of the SVZ stained for BrdU in vehicle-treated (Veh) rats without IR (0 Gy), in vehicle-treated rats after IR (6 Gy), and in CEPO-treated rats (CEPO) without or after IR. Bar = 100 μm. **(B)** There was no difference between the number of BrdU-positive cells in the SVZ of vehicle-treated rats after IR (*n* = 10) and that without IR (*n* = 8). The number of BrdU-positive cells in the SVZ of CEPO-treated rats after IR (*n* = 9) was lower than that of non-irradiated CEPO-treated rats (*n* = 9) (**p < 0.01). Positive cells were counted as described in the Materials and methods section. Results are presented as mean ± S.E.M.

We first examined the effects of IR and/or CEPO on proliferation in the SGZ of the DG and the SVZ. Rats were irradiated on P9 and injected with CEPO 1 day before IR; immediately before IR; and then 2, 4, and 6 days after IR (i.e., on P8, P9, P11, P13, and P15, respectively). To examine proliferation, BrdU was injected 6, 7, and 8 days after IR (i.e., on P15, P16, and P17, respectively), and rats were sacrificed on P24 (Figure 1). Representative photomicrographs of the SVZ and DG in both vehicle- and CEPO-injected rats with or without 6 Gy IR of the ipsilateral hemisphere stained for BrdU are shown in Figures 2A, 3A, respectively. The number of BrdU-positive cells in the SGZ of control rats (vehicle treatment) decreased by >50% after IR (Figure 2B; *p* < 0.01). In CEPO-treated rats, the number of BrdU-positive cells in the SGZ decreased to the same degree after IR (Figure 2B; *p* < 0.01). The number of BrdU-positive cells remained unaffected both in the non-irradiated and irradiated rats.

In vehicle-treated animals, the number of BrdU-positive cells in the SVZ did not decrease after IR (Figure 3B), whereas in CEPO-treated rats, it significantly reduced by 31% after IR (Figure 3B; *p* < 0.01). CEPO did not alter the number of BrdU-positive cells in non-irradiated rats. These results indicate that CEPO decreases proliferation in the SVZ after IR.

### Impact of CEPO on neurogenesis after IR

**Figure 4:**
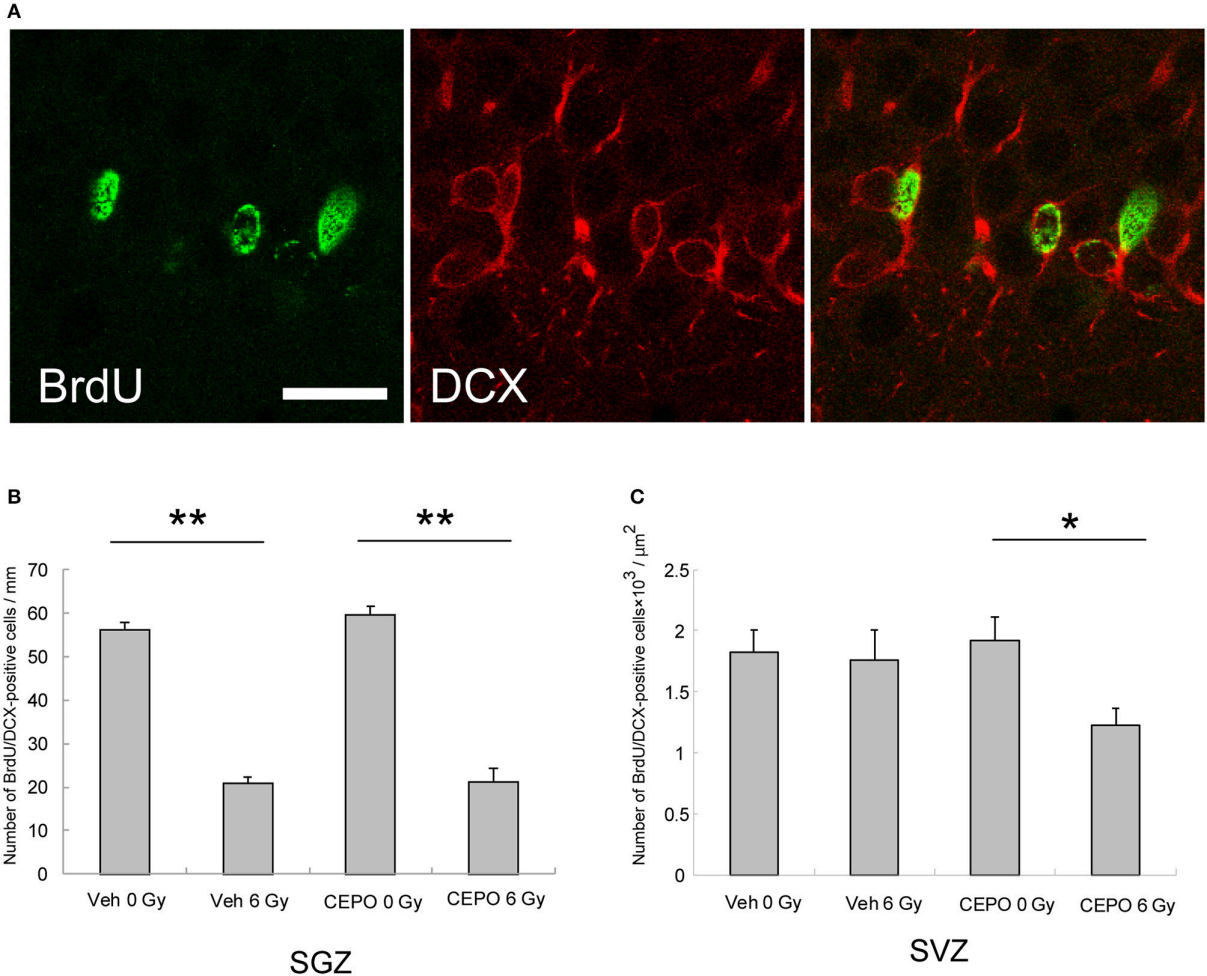
Neurogenesis in the subgranular zone (SGZ) and the subventricular zone (SVZ). **(A)** Representative microphotographs of the SGZ stained for BrdU (green) and DCX (red) 15 days after IR. Bar = 25 μm. **(B)** The number of BrdU/DCX double-positive cells in the SGZ of vehicle-treated rats after IR (*n* = 8) was significantly lower than that of non-irradiated vehicle-treated rats (*n* = 7) (**p < 0.01). The number of BrdU/DCX double-positive cells in the DG of CEPO-treated rats after IR (*n* = 6) was significantly lower than that of non-irradiated CEPO-treated rats (*n* = 9) (**p < 0.05). CEPO did not alter the number of BrdU/DCX double-positive cells in the SGZ, in non-irradiated rats, or in irradiated rats. **(C)** There was no significant difference between the number of BrdU/DCX double-positive cells in the SVZ of vehicle-treated rats after IR (*n* = 10) and that without IR (*n* = 8). The number of BrdU/DCX double-positive cells in the SVZ of CEPO-treated rats after IR (*n* = 9) was significantly lower than that of non-irradiated CEPO-treated rats (*n* = 9) (*p < 0.05). Positive cells were counted as described in the Materials and methods section. Results are presented as mean ± S.E.M.

Next, we examined the effect of CEPO on post-IR neurogenesis. Rats were irradiated and then treated with CEPO, and BrdU was injected as described above. To examine neurogenesis, sections were stained with antibodies to BrdU and DCX, and the BrdU/DCX double-positive cells were counted. Figure 4A presents photomicrographs of the SVZ and DG in both vehicle- and CEPO-treated rats with or without 6 Gy IR to the ipsilateral hemisphere stained for BrdU and DCX.

The number of BrdU/DCX double-positive cells in the SGZ of vehicle-treated rats significantly decreased after IR (58%, *p* < 0.01; Figure 4B). After CEPO treatment, the number of BrdU/DCX double-positive cells in the SGZ decreased to the same degree after IR (Figure 4B; *p* < 0.01). In both non-irradiated and irradiated rats, the number of BrdU/DCX double-positive cells was not significantly affected by CEPO.

In contrast, in vehicle-treated rats, the number of BrdU/DCX double-positive cells in the SVZ did not decrease (Figure 4C), whereas in CEPO-treated rats, there was a 36% decrease after IR (Figure 4C; *p* < 0.05). CEPO administration after IR reduced neurogenesis by 30% in the SVZ compared with that in vehicle-treated rats (Figure 4C), although the same effect was not observed in non-irradiated rats. These results indicate that CEPO decreases neurogenesis in the SVZ after IR.

### Impact of CEPO on microglia after IR

**Figure 5:**
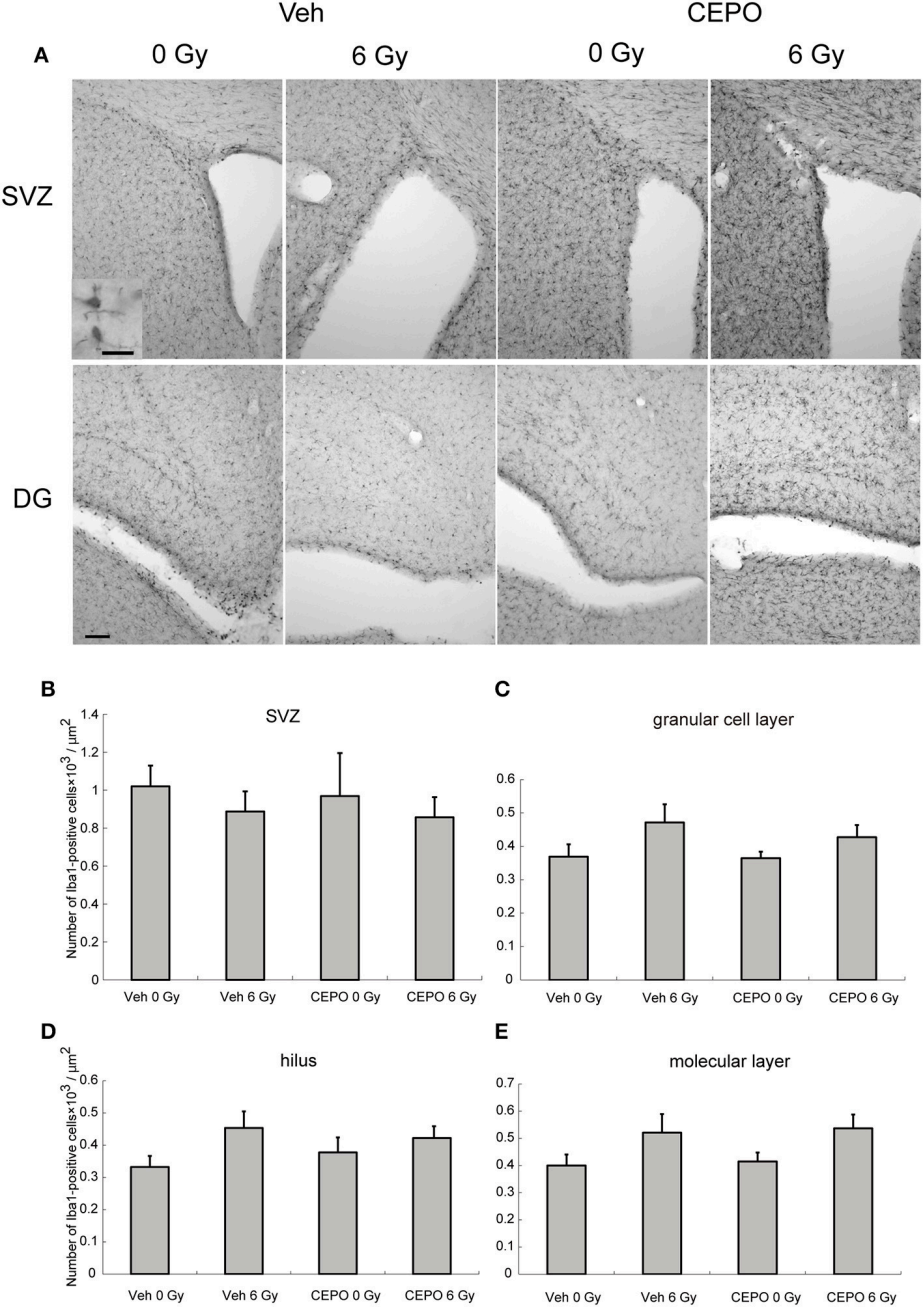
Impact of CEPO on the number of microglia after IR. **(A)** Representative microphotographs of the SVZ and DG of vehicle- (Veh) and CEPO-treated (CEPO) rat brains without (0 Gy) or after IR (6 Gy) stained for Iba1. Bar = 20 (insert), 100 μm. **(B–E)** Quantification of microglia in different regions. There was no difference between the numbers of Iba1-positive cells in the SVZ. **(B)**, GCL. **(C)**, hilus. **(D)**, or ML. **(E)** of vehicle-treated (Veh) rats compared with those of CEPO-treated (CEPO) rats with or without IR (*n* = 5). Positive cells were counted as described in the Materials and methods section. Results are presented as mean ± S.E.M.

Rats were irradiated and treated with CEPO as described above and the number of Iba1-positive cells in the SVZ and DG (GCL, hilus, and ML) was counted 2 weeks after IR. Representative photomicrographs of the SVZ and DG in both vehicle- and CEPO-treated rats with and without 6 Gy IR stained for Iba1 are shown in Figure 5A. The number of Iba1-positive cells was not changed after IR in the SVZ, GCL, hilus, or the ML (Figures 5B–E). No areas of both non-irradiated and irradiated brains were affected by CEPO (Figures 5B-E).

## Discussion

In the present study, we examined the post-IR effects of CEPO on proliferation and neurogenesis in neurogenic regions and demonstrated that CEPO decreased neurogenesis in the SVZ.

The SVZ and DG undergo a decrease in proliferation after IR, both in adult (47, 48) and immature and juvenile (15) rats. In the present study, we demonstrated an IR-induced decrease in the DG, but not in the SVZ, which is in line with our earlier findings in the rat brain where proliferation in the SVZ, but not in the DG, was transiently decreased and subsequently recovered to some extent (36, 49). In neurogenic regions, neurogenesis is decreased after IR both in adult (50) and juvenile and immature (36, 51) brains. Although there is an association between reduced hippocampal neurogenesis and cognitive impairment (15), functional consequences of reduced neurogenesis in the SVZ beyond olfaction are not well studied.

It has been shown that CEPO enhances proliferation *in vitro* (41) and *in vivo* (42) and exerts neuroprotective effects in various models (39). However, in the present study, we did not observe a protective effect on the proliferation rates of the hippocampal SGZ. Moreover, post-IR proliferation in the SVZ was unexpectedly decreased following CEPO treatment. The mechanism underlying this negative effect remains unclear. CEPO, which retains the neuroprotective properties of EPO without exerting an erythropoietic effect, has the potential to be an ideal neuroprotective compound, given its limited toxicity. CEPO has been shown to be protective in various animal models of brain injury (39). CEPO improves renal function and survival in acute kidney injury models without raising hematocrit levels and blood pressure as substantially as EPO (52). Ma et al. indicated that CEPO exerted antiapoptotic activity in myocardial cells independent of JAK2/STAT5 signaling, which was involved in the effect of EPO (53). Also in an IR model, Erbayraktar et al. showed that CEPO reduced brain injury in adult rats after highly localized IR using a gamma-knife (54). However, the dose used in that study was 100 Gy, which is very high and necrotizing, thereby generating a different type of injury than that observed after 6 Gy. In contrast, because CEPO appeared to aggravate the negative effects on proliferation and neurogenesis after a moderate dose of IR, our findings raise concerns regarding the potential use of EPO-related compounds, particularly CEPO, during and after radiotherapy.

Microglia are antigen-presenting scavenger cells that can engulf invading microorganisms, remove deleterious debris, secrete growth factors to promote tissue repair, and maintain tissue homeostasis. However, they can also be transformed into cytotoxic cells (10, 55). It has been shown that the number of microglia is increased in animal models of neurological disorders in both mature (34) and immature (56) brains. However, after IR to the juvenile brain, the number of microglia decreases. In P9 rats, we observed a decrease at 7 days after 8 Gy, which was preceded by an initial increase 6 h after IR (57). During normal brain development, microglia proliferation peaks at P9 (58) and it is conceivable that IR may be deleterious to proliferating microglia, as supported by our findings (57). In the present study, 6 Gy IR to P9 rat brains did not significantly alter the number of microglia in the SVZ or in the hippocampus. This discrepancy between our present and previous findings may be attributed to the lower dose used in the present study (6 vs. 8 Gy). CEPO has been shown to ameliorate the increase in microglia numbers after injury (18, 21, 34); however, we did not find any effect of CEPO on microglia numbers after IR.

There are some limitations of the present study. First, we have not quantified also the number of mature neurons (BrdU+/NeuN+ cells), only the immature neurons (BrdU+/DCX+ cells), so although it is unlikely that the decrease in the SVZ in CEPO-treated rats after IR did not also result in a decrease in mature neurons, this remains to shown unequivocally. Second, for future studies it would be valuable to investigate the dose-response effect by using different doses of CEPO on neurogenesis, since high concentrations of EPO under hypoxic, but not normoxic conditions invoked apoptosis (59). Finally, the underlying mechanisms are unclear and remain to be elucidated.

## Conclusion

EPO has been shown in other paradigms to enhance neurogenesis, but CEPO, which retains the protective properties of EPO but lacks its erythrogenic effects, did not stimulate neurogenesis in non-irradiated or irradiated rat brains. It even aggravated the negative effects of IR on neurogenesis, which raises concerns regarding the use of EPO-related compounds after radiotherapy.

## Author contributions

KO, YS, AO, and MS were actively involved in the experiments. KO, YS, and KB conceived and designed the present study. KO, YS, CZ, ML, HGK, and KB interpreted the data. YS drafted an initial manuscript, which was critically revised by KO, AO, MS, CZ, ML, HGK, and KB. All authors approved the final manuscript as submitted and agree to be accountable for all aspects of the work.

### Conflict of interest statement

The authors declare that the research was conducted in the absence of any commercial or financial relationships that could be construed as a potential conflict of interest.

## Abbreviations

EPO: erythropoietin
CEPO: carbamylated erythropoietin
IR: irradiation
BrdU: 5-bromo-2-deoxyuridine
GCL: granule cell layer
SVZ: subventricular zone
dcx: doublecortin
DG: dentate gyrus
SGZ: subgranular zone
ML: molecular layer.
